# Global Dilemma and Needs Assessment Toward Achieving Sustainable Development Goals in Controlling Leishmaniasis

**DOI:** 10.1007/s44197-024-00190-z

**Published:** 2024-03-11

**Authors:** Mehdi Bamorovat, Iraj Sharifi, Ahmad Khosravi, Mohammad Reza Aflatoonian, Setareh Agha Kuchak Afshari, Ehsan Salarkia, Fatemeh Sharifi, Behnaz Aflatoonian, Faranak Gharachorloo, Ali Khamesipour, Mehdi Mohebali, Omid Zamani, Mohammad Reza Shirzadi, Mohammad Mahdi Gouya

**Affiliations:** 1 Leishmaniasis Research Center, Kerman University of Medical Sciences, Kerman, Iran; 2 Medical Mycology and Bacteriology Research Center, Kerman University of Medical Sciences, Kerman, Iran; 3 Research Center of Tropical and Infectious Diseases, Kerman University of Medical Sciences, Kerman, Iran; 4 Center for Communicable Diseases Control, Ministry of Health and Medical Education, Tehran, Iran; 5 Center for Research and Training in Skin Diseases and Leprosy, Tehran University of Medical Sciences, Tehran, Iran; 6 Department of Medical Parasitology and Mycology, School of Public Health, Tehran University of Medical Sciences, Tehran, Iran; 7 Universal Health Coverage for Communicable Diseases (UHC: CD), World Health Organization, Country Office, Tehran, Iran

**Keywords:** Leishmaniasis challenges, Needs assessment, Development goals, Control strategies

## Abstract

Leishmaniasis is a disease of poverty that imposes a devastating medical, social, and economic burden on over 1 billion people nationwide. To date, no in-depth study to analyze the major global challenges and needs assessment has been carried out. This investigation aimed to explore a comprehensive narrative review of leishmaniasis’s main challenges and initially highlight obstacles that might impede the implementation of control measures. Also, we propose a specific list of priorities for needs assessment. The presence of socioeconomic factors, multiple clinical and epidemiological forms, various *Leishmania* species, the complexity of the life cycle, the absence of effective drugs and vaccines, and the lack of efficient vector and reservoir control make this organism unique and sophisticated in playing a tangled role to react tricky with its surrounding environments, despite extensive efforts and implementation of all-inclusive former control measures. These facts indicate that the previous strategic plans, financial support, and basic infrastructures connected to leishmaniasis surveillance are still insufficient. Strengthening the leishmaniasis framework in a context of accelerated programmatic action and intensification of cross-cutting activities along with other neglected tropical diseases (NTDs) is confidently expected to result in greater effectiveness, cost–benefit, and fruitful management. Sensitive diagnostics, effective therapeutics, and efficacious vaccines are vital to accelerating advancement toward elimination, and reducing morbidity/mortality and program costs. Collective actions devoted by all sectors and policy-makers can hopefully overcome technical and operational barriers to guarantee that effective and coordinated implementation plans are sustained to meet the road map for NTDs 2021- 2030 goals.

## Introduction

Leishmaniasis disproportionately affects inhabitants residing in poverty, mainly in the tropics and subtropics [1, 2]. It imposes a major health, social, and economic burden on over one billion people across the globe, notably in low-income nations and the most deprived groups in middle-income countries [3, 4]. This complex disease has a substantial devastating impact in terms of morbidity and mortality on people living in the affected countries [5]. Such disadvantaged communities lack timely access to affordable therapy in fragile health systems leaving a considerable number severely damaged and disfigured, frequently resulting in social exclusion, discrimination, distress, life-long stigmatization, and serious disability [6].

Leishmaniasis is also a dynamic and intricate complication. Such complexity is further amplified by the lack of approved vaccines, safe and effective drugs [7–9], numerous biological vectors [10, 11], abundant reservoir hosts[12], and diverse ecological habitats. Aside from manifold risk factors, the biology of the organism is sophisticated for possessing a mysterious kinetoplast containing a mitochondrion that plays a tangled role and reacts tricky with its surrounding environmental niches. All these features enable the organism to take advantage of so many precipitating factors and harsh host environment, despite the presence and implementation of a good deal of all-inclusive control measures [13, 14].

**Fig. 1:**
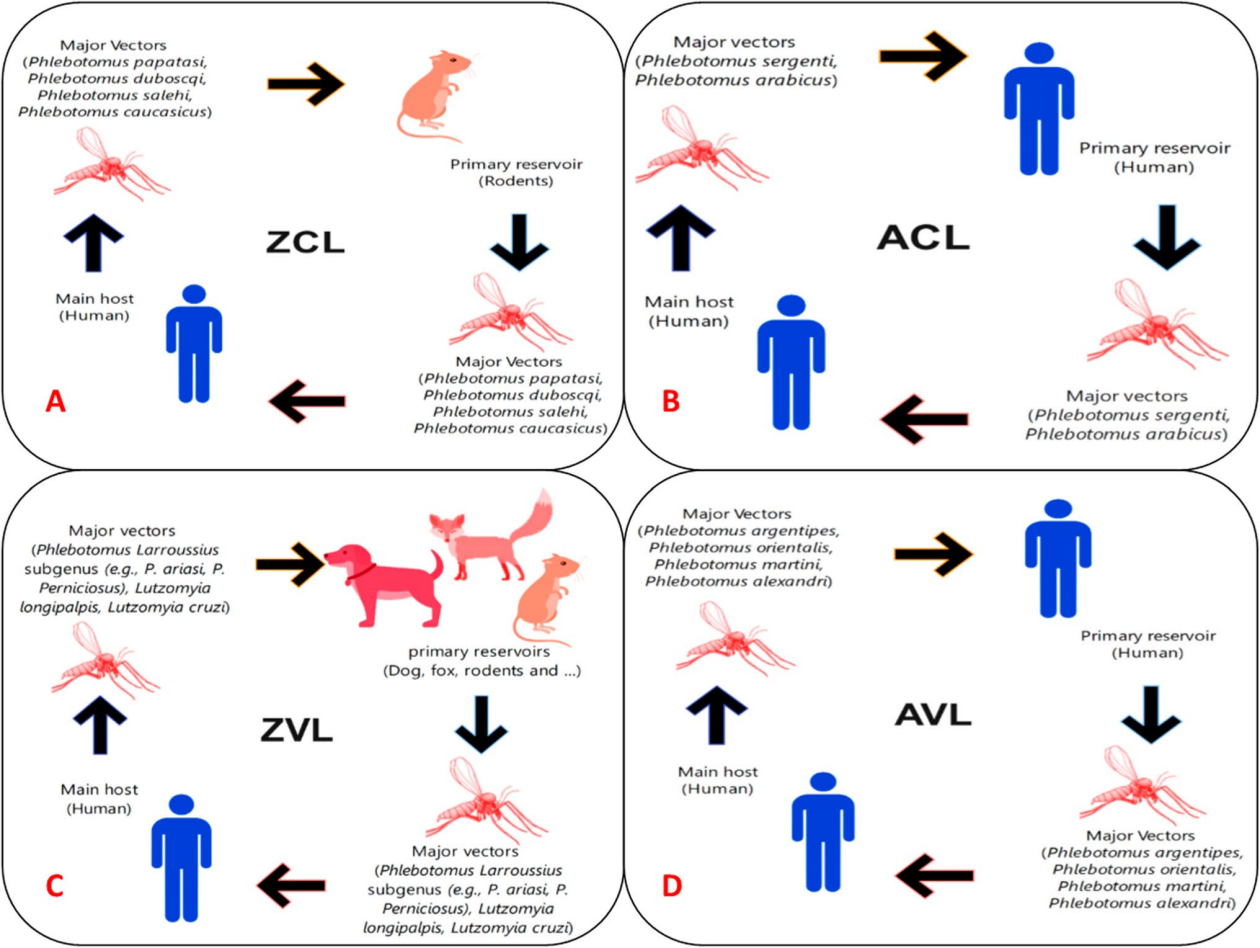
Global distribution of four eco-epidemiological forms of leishmaniasis: **A** zoonotic cutaneous leishmaniasis (ZCL), **B** anthroponotic CL (ACL), **C** zoonotic visceral leishmaniasis (ZVL), and **D** anthroponotic VL (AVL)

Among many clinical presentations and epidemiological forms, two major types of leishmaniasis include visceral (VL), also known as Kala-azar, with mortality potential in over 95% of patients if remain untreated [15]. Currently, approximately, a prevalence of 50,000–90,000 has annually been reported primarily in India, Brazil, and East African countries. While cutaneous leishmaniasis (CL) is the most widespread and main public health illness, responsible for 95% of global cases [16]. Figure 1 shows the global distribution of four eco-epidemiological forms of leishmaniasis. Around 82% of CL cases happen in the Eastern Mediterranean Region and the remaining in the Americas, Africa, and Europe. The number of CL cases reported to the World Health Organization (WHO) is estimated at 0.6–1 million annually, although this figure is a fraction of the actual number [3, 17].

The leishmaniasis needs assessment is compatible with the despairing necessity for well-organized evidence on the control database worldwide [18]. We have also abridged significant challenges and crucial requirements for leading an initial control scheme in the direction of the leishmaniasis elimination plan. Besides, a professional team including skilled health and clinical academics allied with the health services and universities was fixed. During a two-year effort, over 23 assemblies were detained composed of extensive assessments, meetings, official visits, and group discussions of numerous issues. This study aimed to explore a thorough narrative review of leishmaniasis’s main challenges and initially highlight obstacles that might impede the implementation of control measures and the efficiency of interventional approaches. Lastly, we propose a specific list of priorities for needs assessment.

## Research Method

**Fig. 2:**
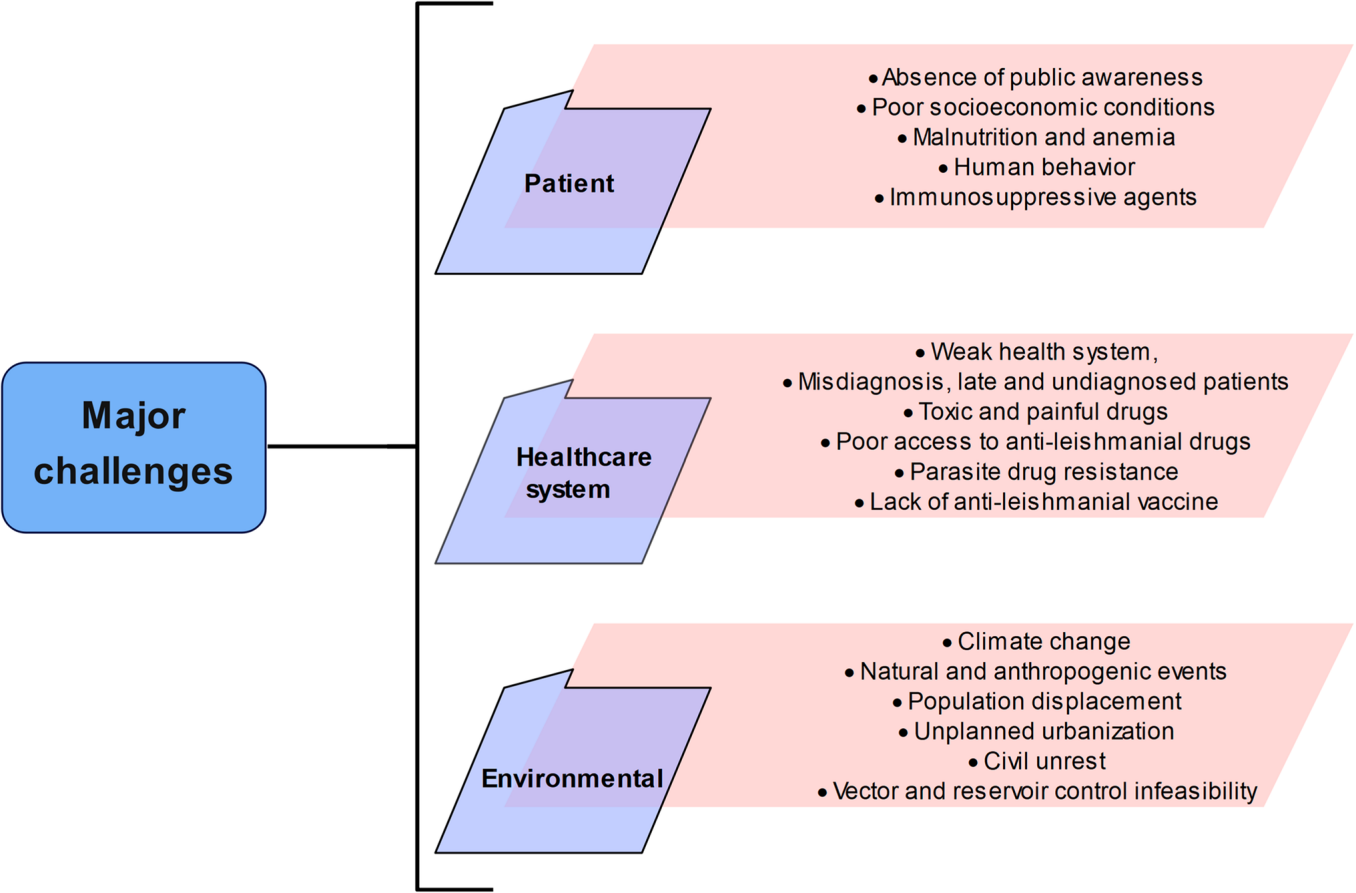
Significant challenges for achieving sustainable development goals (control, elimination, and eradication)

A widespread literature search was conducted using Google Scholar, PubMed, and MEDLINE databases. To assess the main challenges and needs, a literature review, research articles, books, and reports were investigated by the following keywords: “leishmaniasis challenges”, “control”, “needs assessment”, “leishmaniasis control”, “leishmaniasis”, “elimination”, “prevalence”, “cutaneous leishmaniasis”, “CL patients”, “VL patients”, “treatment”, “health system”, “treatment failure”, “*Leishmania”,* and “meglumine antimoniate”. Figure 2 shows the major challenges for eliminating leishmaniasis. Therefore, in this study, the effective and interactive challenges and needs assessment in eliminating, and controlling leishmaniasis are reviewed, described, and discussed for ending the neglect to accomplish sustainable development goals.

## Major Challenges

### Patient Challenges

#### Absence of Public Awareness

Lack of knowledge and appropriate health information among local inhabitants in endemic areas where the disease is prevailing significantly reduces the efficiency of reservoir host and vector control programs [19–21]. To increase the public knowledge about the disease and the effectiveness of patient management, proper control interventions must be attained through improved awareness about the disease among the general communities and active involvement of the local population in control activities [21, 22]. Prevention and therapeutic measures are fully reliant on a detailed knowledge of the domestic and wildlife cycle [23].

#### Socioeconomic Conditions

Leishmaniasis is a poverty-related disease. Poor housing conditions and the absence of sewage and garbage management may enhance sandfly resting and breeding places and facilitate human contact. Sandflies are interested in unsanitary and crowded housing conditions around domestic dwellings where they bite and feed on human blood and transmit the disease [11, 21, 24–26].

#### Malnutrition and Anemia

Lack of vitamin A, iron, zinc, and protein-energy malnutrition execrable the infection resulting in a full-scale disease condition [27]. The severity of leishmaniasis can be intensified due to malnutrition [28]. Nourishment also disturbs the host and the preference of the sandfly to bite a particular host [29].

#### Human Behavior

Human behavior is directly linked with leishmaniasis burden. Sleeping on the ground and outside houses in impoverished endemic communities has the potential to increase the risk of the affliction of the disease[15, 30]. In this circumstance, people come in contact with the bite of sandflies and become infected. This situation further causes people to leave their original homes and become exposed to vectors and contract the disease. Sandflies are readily attracted to unprotected and crowded housing because it is easier to access their body and feed on their blood [29, 31].

#### Immunosuppressive Agents

Both human immunodeficiency virus (HIV)/acquired immunodeficiency syndrome (AIDS)-related and nonrelated infections and noninfectious immunosuppression agents pose significant challenges to the management of CL and VL. Although the actual burden of immune-suppressed patients coinfected with leishmaniasis is possibly underestimated. The burden of concerted HIV/AIDS-related and non-HIV/AIDS-related is raised due to travel into endemic areas. Regarding clinical presentation, atypical manifestation has been reported in active VL super-imposed HIV-infected patients [32]. High coinfection prevalence is reported in Brazil, Ethiopia, and the state of Bihar, India [31]. Most of these super-infected patients face greater failure and even death [33]. Treatment failure is a prevalent phenomenon in leishmaniasis cases coinfected with diabetes, opium addicts, and those patients who use immunosuppressive drugs. They often acquire the severe form of the disease and become chronic and unresponsive to conventional drugs [7].

HIV remains a major national, regional, and global public health challenge. Approximately 39 million people living with HIV in 2022. Leishmaniasis-AIDS co-infection can present itself as CL or VL. Nonetheless, in HIV/AIDS patients, low-sensitivity serological tests make a definite diagnosis of cases troublesome [31, 34]. Mixed infection between leishmaniasis and HIV/AIDS in people living in tropical countries where these two infections are endemic frequently occurs. There have been reports of *Leishmania*-HIV co-infection from 45 countries as of 2021 [31, 35].

In addition, the role of asymptomatic carrier cases with VL is poorly perceived. Comparing immunocompetent patients to the number of apparent VL patients as demonstrated by serological and other intrinsic data, its proportion is five to ten times greater [36]. Therefore, medications accessible to treat both CL and VL are more restricted and lead to prominent adverse effects. Moreover, in patients infected with HIV/AIDS, these unfavorable outcomes are more notable, and relapses and fatalities are recurring. As a result, such simultaneous concurrence which often occurs in developing countries remains challenging [34]. VL negatively affects responses to entire antiretroviral therapy and co-infected patients are difficult to cure, especially when their CD4 cell count is < 200 cells /mm^3^ [35]_._

#### Leishmaniasis and Other Infectious Diseases

Multiple infections among leishmaniasis and other infectious diseases, notably in endemic areas are common. It depends on the geographical peculiarities, environmental factors, vector accessibility, and host-parasite interaction [37]. Leishmaniasis, remarkably VL is an immunosuppressive illness that enables opportunistic microbial and parasitic infections to coexist. Mixed infections with tuberculosis, leprosy, malaria, schistosomiasis, and other invading agents have often been reported [38–40]. In most co-infections, the disease severity frequently progresses to a fulminating form and leads to a high mortality rate.

### Healthcare System Challenges

#### Weak Health System

In underdeveloped countries, healthcare services are poorly delivered. Available facilities are understaffed with inadequate resources and often incapable of offering appropriate and acceptable healthcare services [4, 21, 41]. Therefore, poor-quality services bring about uncertainty and discourage people from looking at healthcare requests. In many health systems patients have to pay fees for general healthcare services to block the subsidy gap [18, 41].

#### Misdiagnosis, Late and Undiagnosed Patients

Leishmaniasis mimics a spectrum of disease conditions, infections, and noninfectious disorders including fungal, viral, bacterial, and parasitic infections, lupus vulgaris, sporotrichosis, tuberculosis, mycobacterial ulcers, zoster, herpes-like and wart viruses, cutaneous diseases, myiasis, tropical ulcers, ecthyma, foreign-body granuloma, acute furunculosis, and skin carcinoma [21, 42, 43]. Precise knowledge of these presentations and confirmation of the etiological agent is highly essential for selecting the proper treatment modality in endemic foci [13, 25, 42–44].

In VL, diagnosis is performed by a combination of clinical manifestations along with parasitological or serological tests. People assumed of suffering from VL must receive the drug of choice immediately [45]. Although in CL and mucocutaneous leishmaniasis (MCL) serological tests have limited value and often clinical presentation with parasitological examinations identify the disease. Owing to the low sensitivity of some of the assigned tests these diseases are not timely detected; therefore, patients serve as a reservoir to perpetuate the organism, regularly become refractory, and remain undiagnosed resulting in serious consequences [25, 46]. The majority of patients who receive treatment late become non-responsive and do not respond properly to conventional therapy [7, 25].

#### Toxic and Painful Drugs

Most conventional formulations including meglumine antimoniate (Glucantime®) and sodium stibogluconate (Pentostam®) and alternative medicines like liposomal amphotericin B (AmBisome®), pentamidine, allopurinol, paromomycin, and azole derivatives are associated with serious adverse effects [7, 42, 47]. Besides, they are applied parentally, are often painful, and induce parasite resistance. Application of these drugs by patients often faces treatment failure and further results in chronicity and exacerbation of the disease condition [4, 7, 48, 49]. Poor treatment adherence is a widespread and ignored phenomenon in the proper treatment of leishmaniasis [4, 21, 50, 51]. At present, control of leishmaniasis depends mainly on chemotherapy. As the drugs of choice are associated with adverse effects, a great number of patients preferred not to receive the drug especially patients with CL [4, 18]. The capability of patients to receive treatment regimens is faced with numerous barriers that include doubt about the efficacy, side effects, work constraints, feeling sick, forgetfulness, living in remote areas, and complex treatment regimens [4, 13, 52–54].

#### Poor Access to Anti-Leishmanial Drugs

First and second-line leishmaniasis drugs are often expensive and unaffordable for control programs and also for individual patients, especially in poverty-stricken nations. Drug affordability can be relatively improved by negotiating with pharmaceutical companies to subsidize the price or possibly donating medicine to some low-income countries [21, 55, 56].

#### Parasite Drug Resistance

Decline or absence of response to a particular drug against ongoing standard drugs through molecular mechanism is a well-known event in treating leishmaniasis [57]. Drug resistance is a basic determinant in leishmaniasis drug failure. *Leishmania* species are unique for their remarkable genomic plasticity and readily undergo genetic mutations in producing drug-resistant genotypes a mechanism allowing them to survive under drug pressure[58]. Monitoring drug resistance to *Leishmania* mutants is currently a challenge as there is no validated and simple phenotypic and genotypic assay to routinely monitor resistance in the field [48, 59]. The phenomenon of drug resistance has notably been a major challenge in anthroponotic leishmaniasis; AVL and ACL due to *L. donovani and L. tropica,* respectively in the Old World where human-to-human transmission or resistant genotypes frequently occur [51, 60–62]. Combination therapy using multiple drugs is considered to be contemplated with variable response outcomes [63–65].

#### Lack of Anti-Leishmanial Vaccine

The history of anti-leishmanial vaccines goes back to the preceding decades [18]. The following key obstacles and restrictions to the development of an effective vaccine against different types of leishmaniasis include: i. The poorly understood means of host − parasite interaction and the elaborateness of the immune reaction accompanying *Leishmania* parasites. ii. Appropriate immunity for the development of a vaccine against different leishmaniasis forms represents a further major issue (CL, MCL, and VL). iii. Lack of knowledge of the numerous factors that could lead to such responses. iii. Unavailability of reliable techniques and methods for evaluating the efficacy of vaccinations. v. The absence of right laboratory animal prototypes for assessing the efficacy of vaccines before they are used in humans and vi. Deficiency of effective delivery methods and appropriate adjuvants to trigger a protective immune response [66].

### Environmental Challenges

#### Climate Change

Leishmaniasis is significantly affected by climatic conditions. The impact of global warming remarkably drought, increased temperature, and decreased precipitation substantially affect the dispersion of leishmaniasis through vector abundance [67]. Climate variations also force people to migrate and leave their homeland to new areas of main municipalities, often in the outskirts where economic and sanitary situations are poor [68, 69]. This condition can expedite the transmission of vector-borne diseases (VBDs) particularly leishmaniasis escalate hotspots of diseases in towns and cities and consecutively facilitate the transmission of emergent infections [67, 70].

#### Natural and Anthropogenic Events

Disasters, including earthquakes, tsunamis, and floods provide numerous precipitating factors and in turn, prepare suitable breeding conditions for the propagation of vectors and spread of parasites to susceptible hosts [71, 72]. Large epidemics describe the disease in greatly populated cities particularly peri-urban inhabitants and disaster-affected public, enhance significantly population motility, frequent wildlife, and animal foodstuffs, and zoonotic diseases between countryside and municipal communities[73]. The incidence of leishmaniasis is highly affected by man-made modifications and natural disasters [74, 75].

#### Population Displacement

Population movement, migration, and the formation of innovative peri-urban communities in the vicinity of endemic areas are the main confounding factors for leishmaniasis. Epidemics of the disease frequently occur when non-immune individuals arrive at the local site where the force of transmission is high [69]. Population movements are significantly accompanied by the spread of neglected tropical diseases (NTDs) and are frequently worsened by deprived health facilities and inadequate medical substructures. Leishmaniasis is a decent example of the penalties provoked by population flexibility [76, 77]. Migration, impoverished people in suburbs of municipalities, and trans-border activities are typical determinants for evolving anthroponotic CL (ACL) outbreaks [71, 73].

#### Unplanned Urbanization

Urbanization implies the population relocation from rural to urban areas, a phenomenon that abandons or diminishes the proportion of such local communities and rapidly expands towns and cities often in emergent nations. Unexpected urbanization is considered a major risk factor for leishmaniasis [78–81]. The incidence of leishmaniasis is often increased because of desertification, deforestation, and human intrusion into the woodland and forest and the transformation of this valuable natural resource into an unintended urbanized area [73, 82, 83].

#### Civil Unrest

Cutaneous and visceral leishmaniases are tightly associated with rising levels of conflict among nations. Particularly, warfare can perform as an empirical proxy for incidental and associated processes of social deterioration, instability, and population displacement and consequently the emergence of leishmaniasis [84, 85]. In the last decade, because of the ongoing war in Syria, Libya, Afghanistan, Yamen, and neighboring countries, the burden of CL has substantially increased in war-affected people [14]. Consequently, masses of displaced people such as Syrian residents left their homeland and crossed the border into Turkey and adjacent countries, causing severe public health and economic effects in these countries, and then motivated toward European countries [85, 86]. It is assumed that about 3 to 4 million people from Afghanistan live in Iran because of prolonged warfare, as the result of social and political instability in their homeland [87].

#### Vector and Reservoir Control Infeasibility

*Leishmania* species are maintained by manifold hosts (some 70 animal species, including humans) consisting of seven mammal orders including Primata, Carnivora, Rodentia, Marsupialia, Cingulata, Pilosa, and Chiroptera – accountable for maintaining *Leishmania* parasites in the domestic and wildlife habitat. The only confirmed vectors of human disease are species and subspecies of *Phlebotomus* and *Lutzomyia* in the Old World and the New World, respectively [23, 31]. About, 600 species of phlebotomine sandflies are recognized but only 60 (10%) of these serve as disease vectors. The control of leishmaniasis depends on multiple factors including the form of the disease, parasite species, geographic location, vector and reservoir dispersion, and density. Chemical and environmental management have so far been neither practical nor eco-friendly [18, 21, 31].

## Needs Assessment

**Fig. 3:**
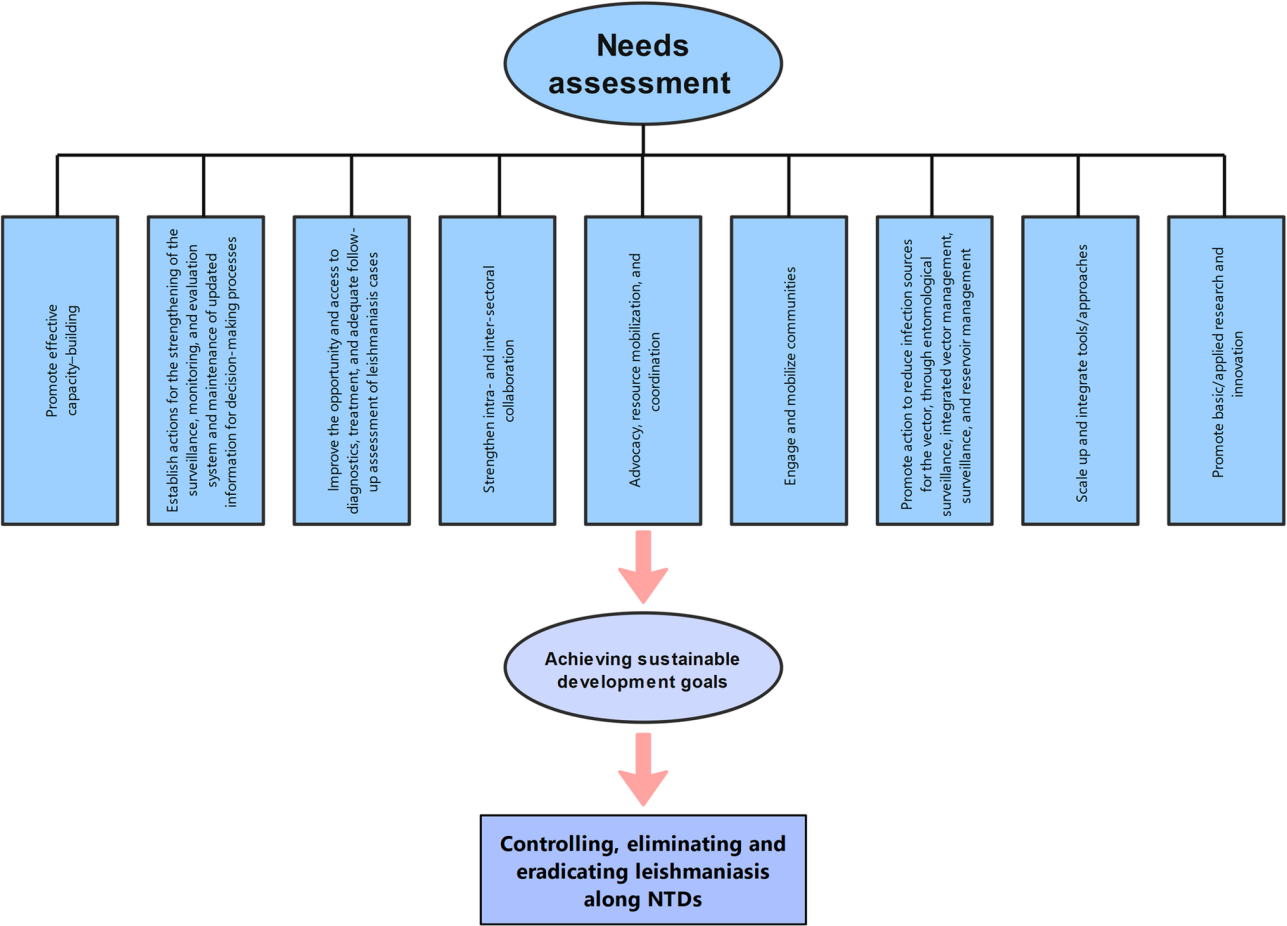
Needs assessment for achieving sustainable development goals in leishmaniasis. Extensive and long-term integrated efforts are desperately needed to attain the above targets through cross-cutting activities and accelerating programmatic actions to control, eliminate, and eradicate neglected tropical diseases (NTDs) including leishmaniasis. The plan requires necessary resources to prepare the infrastructure, and the required costs for the implementation of long-lasting preventive and therapeutic measures including the diagnostic kits, essential drugs, and personnel expenses for coordinating, integrating, mainstreaming, and strengthening health systems to leishmaniasis interventions across health and nonhealth sectors

National programs should develop strategic objectives that are in line with the epidemiology of leishmaniasis, the capability of the health system, the available resources, and the need for interventions to achieve continuous effective coverage of leishmaniasis services. This is similar to how comprehensive multi-year plans for the control of diseases, such as NTDs master plans, are designed [5, 88]. The NTDs framework directs program planners to place these objectives in their proper perspective and to move toward the cross-cutting strategies that are promoted in the road map [4, 89]. Eliminating leishmaniasis to attain sustainable development goals requires a lot of commitment, extensive ongoing efforts, and providing the necessary resources to prepare the infrastructure for integrating, mainstreaming, coordinating, and strengthening health systems to leishmaniasis interventions across health and non-health sectors. Figure 3 displays the needs assessment and activities for reaching sustainable development targets in leishmaniasis.

### Promote Effective Capacity Building

Strengthening training for health staff including physicians, parasitologists, epidemiologists, entomologists and other relevant personnel involved in the leishmaniasis control plan is of great importance.

i. National guidelines for leishmaniasis diagnosis, treatment, and control should be updated and published.
ii. Religious, political, and community leaders should be sensitized and educated to cooperate in various aspects of the program.
iii. Public awareness-raising sessions about potential risk factors linked with the disease should be conducted.

### Establish Actions for the Strengthening of the Surveillance, Monitoring, and Evaluation System and Maintenance of Updated Information for Decision-Making Processes

Surveillance and monitoring are crucial to evaluate each component of the program to measure its effectiveness and advancement to achieve objectives.

i. Enhancing active case detections along with passive case-finding by the health surveillance personnel, volunteers, and community representatives.
ii. Surveillance and monitoring activities by staff and health workforces for the specific task regularly.
iii. Logical collection, reporting, analysis, and explanation of public health-associated data for the policy-makers and health authorities to assess, improve, and implement public preparation programs.
iv. Establishing a common electronic registry and backup data system.
v. Surveillance, monitoring, and evaluation of the disease indicators (i.e., suspected lesions for CL and fever for VL) by health personnel for early detection and proper treatment modality.
vi. Regular surveillance and monitoring of drug unresponsiveness (i.e., resistance) and efficacy of insecticides by bioassay tests.
vii. Surveillance, monitoring, and evaluation of people’s behaviors using insecticide-impregnated bed nets.
viii. Updating checklists for registering, recording, and reporting.

### Improve the Opportunity and Access to Diagnostics, Treatment, and Adequate Follow-Up Assessment of Leishmaniasis Cases

Proper diagnosis (i.e*., Leishmania*), identification (i.e., down to species level), effective therapy, and adequate follow-up assessment of leishmaniasis are essential for any effective control program.

i. Application of rapid sensitive diagnostic tests (RDT) should be in high priority.
ii. Follow-up examination of the leishmaniasis cases ensuing treatment until recovery is critical.
iii. Accessibility to health clinics in endemic areas is critical.
iv. Availability of standard diagnostic reagents, kits, and first-line drugs in the endemic foci should be of extreme priority.
v. Early detection and effective and prompt treatment of patients with ACL and anthroponotic (AVL) should be of high priority.
vi. Encouraging pooled procurement mechanisms for health and clinical where required.

### Strengthen Intra- and Inter-Sectoral Collaboration

i. Inviting different representatives of the health sectors to participate in the provincial health council for coordination and collaboration with the leishmaniasis control program in future planning.
ii. Reinforcing the political commitment of partner ministries through the Centers for Disease Control and Prevention (CDC) and Ministry of Health and Medical Education (MOHME) by validating the cooperation in a memorandum of understanding (MOU).
iii. Raising the control plan in the MOHME deputy council for intra-sectoral sensitization and cooperation.
iv. Presenting the control program in the government cabinet for inter-sectoral collaboration of the ministries.
v. Inviting relevant customers and stakeholders to a meeting at the district level to be familiar with the leishmaniasis control program and seeking their support for the plan.
vi. Coordination with municipalities through CDC/MOHME and the Ministry of Interior Affairs to raise the social, health, and medical importance of such control program to take strong action in solid waste management, promotion and overall cleaning of the cities, and controlling the population of stray dogs.
vii. Inviting the neighboring country authorities to sign an MOU agreement to collaborate, coordinate and activity and/or passively monitor trans-border movement.

### Advocacy, Resource Mobilization, and Coordination

Advocacy and resource mobilization are essential elements of a successful control program to assure new and supplementary funding, manpower, and other resources to facilitate the efforts and increase its sustainability.

i. Develop a coherent and dynamic resource mobilization plan to initiate and enhance program activities.
ii. Promote adequate funding and support for the achievement of the program at the country, provincial, district, and community levels.
iii. Enlarge the funding base of partnership contributions.
iv. Achieve effective organizational arguments in support of resource mobilization.
v. Provide leishmaniasis stakeholders with an advocacy strategy and implementation guide to assist them with mobilizing resources for leishmaniasis control in endemic areas.
vi. Build advocacy, communication, and social mobilization at national and sub-national levels.
vii. In addition to the provision of resources by the government, additional financing should be secured for development activities and research relevant to the control and treatment of the disease by donors (private and non-governmental organizations (NGOs)).

### Engage and Mobilize Communities

The plan for leishmaniasis control should be enriched and potentiated with public households as full and equal associates in different phases of the plan.

i. Establishing and introducing health volunteer forces (NGOs and private) to take an active part in reducing physical, environmental, social, and structural inequality via the active participation of community members, organizations, and leaders.
ii. Promoting community engagement and mobilization through political authorities and religious leaders in endemic foci.
iii. Seeking community participation and sensitization through social media (T.V., handouts, poster education, cyberspace, and the press).
iv. Promoting a healthy lifestyle and health policies through health volunteers, health personnel, and social media.
v. Paying special attention to immigrants from endemic countries in encouraging their awareness about the disease.

### Promote Action to Reduce Infection Sources for the Vector, Through Entomological Surveillance, Integrated Vector Management, Surveillance, and Reservoir Management

Systematic entomological and reservoir surveillance is highly necessary for any successful leishmaniasis control program.

i. Incriminating the vector fauna and reservoir species is fundamental for designing effective leishmaniasis intervention.
ii. Determining the peak seasonal activity of sandflies in the endemic foci is vital to controlling the disease.
iii. Setting up a sentinel site for regular surveillance of leishmaniasis is critical.
iv. Evaluation of insecticide-impregnated bed nets and environmental insecticide spraying is necessary for planning and evidence-based decision-making.
v. Evaluation of different poisons and biological measures for rodent control is needed.
vi. Periodical bioassays for controlling the emergence of insecticide resistance are extremely important.

### Scale Up and Integrate Tools/Approaches

Refining the quality of trained attendance by implementing good clinical practice and evidence-based guidelines (i.e., good laboratory practice, GLP; standard operating procedure, SOP; and good manufacturing practice, GMP) in national policy and protocols.

i. Scaling up evidence-based practices, tools, and approaches in teamwork with national partners.
ii. Improving delivery and access to a health product, and mechanisms to legalize the quality, safety, and effectiveness of medicines, consistent with GMP and active supply chain supervision, are serious components of a well-functional health network related to leishmaniasis and other infectious disease control strategies.
iii. Several issues contribute to the price of medical products, and health policies should address these aspects to enhance their economy and availability through competition of prices, etc. The health authorities in the country should monitor supply and distribution chains and procurement practices carefully to diminish expenses that could unfavorably affect the price of these products.

### Promote Basic/Applied Research and Innovation

i. Holding applied and basic research workshops by the universities and health surveillance services at county and provincial levels for the health staff and physicians to improve and strengthen their research capabilities.
ii. Providing a list of research priorities and needs relevant to leishmaniasis.
iii. Allocation of pre-determined research grants as incentives for those who are willing to conduct basic/applied research relevant to leishmaniasis.
iv. Establish and strengthen mechanisms to improve ethical review and regulation of the quality, safety, and efficacy of healthcare products and medical devices, where appropriate.
v. Mapping research and development to identify gaps in research and development on leishmaniasis.

## Brief Discussion and Conclusion

Eliminating leishmaniasis to achieve sustainable development goals requires a lot of commitment and widespread ongoing efforts. The plan requires necessary resources to prepare the infrastructure, and the required costs for the implementation of long-lasting preventive and therapeutic measures, including the diagnostic kits, essential drugs, and personnel expenses for coordinating, integrating, mainstreaming, and strengthening health systems to leishmaniasis interventions across health and non-health sectors. Governments and stakeholders have made significant progress toward achieving the leishmaniasis targets and milestones over the past decades, despite tremendous challenges and gaps. However, sustaining and accelerating these goals and attaining the targets for control, and overall elimination of leishmaniasis needs additional efforts, effective approaches, a comprehensive plan, and on-top financial support.

The control program requires the ‘one health’ approach to bring together stakeholders in relevant sectors to implement policies and programs for the sustenance of public health results. For leishmaniasis, this strategy supports an integrated and multisectoral coordinated approach to the parasite species, disease hosts, phlebotomine vectors, and environmental factors, with the clear and precise assignment of roles and responsibilities. The one health approach is critical to sustain integrated interventions against the disease as it can leverage existing infrastructure to support surveillance, clinical management, vector control, animal husbandry interventions, and data-sharing among relevant sectors. By engaging with one health committee, working groups, and consultation and guidance of WHO, national leishmaniasis programs along with local health authorities can analyze and improve their systematic data collection, documentation, and management, and strengthen the implementation of control strategies to support sustained effective coverage of overall services.

## Data Availability

All data generated or analyzed during this study are included in this published article.

## Abbreviations

VL: Visceral leishmaniasis
CL: Cutaneous leishmaniasis
WHO: World Health Organization
MCL: Mucocutaneous leishmaniasis
HIV: Human immunodeficiency virus
AIDS: Acquired immunodeficiency syndrome
VBDs: Vector-borne diseases
NTDs: Neglected tropical diseases
ACL: Anthroponotic cutaneous leishmaniasis
RDT: Rapid sensitive diagnostic tests
MOHME: Ministry of Health and Medical Education
MOU: Memorandum of understanding
CDC: Centers for Disease Control and Prevention
NGOs: Non-governmental organizations
GLP: Good laboratory practice
SOP: Standard operating procedure
GMP: Good manufacturing practice
